# Factors Associated With Inaccurate Recall of Inherited Cancer Genetic Test Results Among Individuals With Germline Pathogenic Variants

**DOI:** 10.1002/cam4.71689

**Published:** 2026-03-07

**Authors:** Deborah L. Cragun, Brittany F. Sears, Karishma Prakash Bharwani, Anne E. Weidner, Jason W. Beckstead, Tuya Pal

**Affiliations:** ^1^ University of South Florida Tampa Florida USA; ^2^ Tampa General Hospital, Cancer Institute Tampa Florida USA; ^3^ Vanderbilt University Medical Center Nashville Tennessee USA

**Keywords:** cancer risk, cancer risk management guidelines, genetic testing, pathogenic variant

## Abstract

**Background:**

Germline pathogenic and likely pathogenic variants (GPV) in cancer susceptibility genes may guide medical care. However, inaccurate recall of test results by patients may impact the uptake of appropriate medical care, underscoring the importance of accurate recall of results.

**Methods:**

Among individuals with a confirmed cancer susceptibility GPV that alters cancer risk management, individuals were categorized based on their accuracy of recall. Multiple logistic regression was performed to determine demographic and psychosocial factors associated with recall of test results.

**Results:**

Of 807 participants, 91 (11%) failed to accurately recall their test results, including 22 who did not remember being tested and 69 whose cancer risk management could be impacted due to inaccurate recall of test result details. Compared to those with accurate recall of test results, those who failed to accurately recall their results were significantly more likely to have a history of cancer [OR = 2.24, *p* = 0.009] and lack a college degree [OR = 2.42, *p* < 0.029]. Additionally, based on standardized survey responses, failure to accurately recall was associated with positive feelings about their results [OR = 1.73, *p* < 0.001], lower emotional distress [OR = 0.65, *p* = 0.004], lower knowledge about hereditary cancer [OR = 0.67, *p* < 0.001], and lower satisfaction with family communication and support related to testing [OR = 0.62, *p* < 0.001]. Participants with GPVs in high‐risk or moderate‐risk breast cancer genes were substantially less likely to demonstrate inaccurate recall (both OR = 0.18, *p* < 0.001) compared to those with GPVs in other actionable cancer genes.

**Discussion:**

Given 11% of participants failed to remember being tested or failed to accurately recall their result, this highlights the importance of documentation and accessibility of test results by both patients and providers to ensure patients receive appropriate care.

## 1 Introduction

Individuals who have a germline pathogenic or likely pathogenic variant (GPV) in an inherited cancer susceptibility gene are at increased risk of developing cancer. For GPVs in many of these genes, strategies exist for cancer risk management (CRM) to detect cancer early or prevent cancer altogether [1, 2]. CRM recommendations vary based on which hereditary cancer susceptibility gene contains a GPV, and accurate recall of test results could potentially impact the receipt of appropriate care. In contrast to GPVs, variants of uncertain significance (VUS) in cancer genes are generally not used to guide CRM [3] unless the variant is upgraded to a GPV in the future.

Accurate recall of information following a medical visit is often critical for patients to adhere to treatment and recommended medical management [4]; however, prior studies suggest that only about 20%–60% of information discussed in medical appointments is recalled and half of the information is recalled incorrectly [4, 5, 6, 7]. Furthermore, inaccuracies can be compounded as information is shared with at‐risk relatives [5]. Patients may even forget they have undergone genetic testing, with a prior study finding that 30% of patients with abnormal tumor and/or germline testing failed to recall undergoing the testing [8]. Even among those who remembered completing testing, over 40% failed to correctly remember their germline or somatic results [8]. That study did not report on factors that impacted recall of test results. However, other studies have found that recall of cancer risk decreases with older age, poorer prognosis, lower health literacy, and less education [6, 7, 9]. Large studies focused on recall of test results and associated psychosocial factors among patients in whom medical care would be impacted are lacking. Thus, we sought to characterize inaccurate recall among those with a GPV in an inherited cancer gene and identify factors associated with inaccurate recall.

## 2 Materials and Methods

### 2.1 Study Design and Participants

Individuals were recruited through the Inherited Cancer Registry (ICARE) [10], the Tennessee State Cancer Registry, and the Florida State Cancer Registry as previously described [11]. Individuals were asked to review the informed consent form, sign an authorization for release of medical records, and complete a baseline survey as part of the parent IMProving Care After Inherited Cancer Testing (IMPACT) study [11]. The current analysis is based on cross‐sectional data from participants who completed the baseline survey and had a medical record‐verified GPV in at least one of 21 selected inherited cancer risk genes, all of which would impact CRM recommendations per the NCCN guidelines and demonstrated risk concordant interpretation across laboratories (per ClinVar and/or CanVar websites). Additional inclusion criteria were: (1) aged 18 years or older at the time of study participation; (2) English‐speaking; (3) had an email address on file; and (4) residing in the United States. Exclusion criteria included those who were not yet at the age for which CRM is recommended. The study was IRB‐approved through Vanderbilt University (IRB #202215), the Tennessee Department of Health IRB (IRB# 2021‐0281), and the Florida Department of Health IRB (IRB# 2023‐022‐VBU), and researchers adhered to ethical guidelines for human subjects research. Recruitment for this study occurred from mid‐December 2021 through mid‐October 2024.

### 2.2 Measures

The baseline survey collected information about receipt of genetic testing. For those who selected “I have already had genetic testing for inherited cancer,” the self‐reported gene and type of result (e.g., positive for a GPV, negative, or VUS) were also collected if known. Demographic and clinical information included highest level of education, income level, insurance status, race/ethnicity, sex at birth, and personal history of cancer. Ten questions assessed genetic testing knowledge with response options of “Agree (Yes)”, “Disagree (No)”, or “I don't know (Unsure)”, and an overall knowledge score was calculated by assigning one point for each correct answer and adding points across all items [12], a 3‐item Likert‐type measure was used to assess health literacy (*α* = 0.48) [13], and a 3‐item Likert‐type measure was modified to assess cancer worry in general, rather than limiting it to breast cancer (*α* = 0.78) [14].

Additional questions in the baseline survey included the multidimensional impact of cancer risk assessment (MICRA) [15], a validated, 21‐item assessment of emotional impacts of genetic testing. Because minor modifications in wording were made to a few MICRA items, exploratory factor analysis was conducted to evaluate construct validity and then inter‐item reliability was calculated for each of the resulting subscales. The distress subscale was created by averaging all six distress items because these loaded together on a single factor (*α* = 0.90). The uncertainty subscale was created by averaging three of the uncertainty items that loaded together on a single factor (*α* = 0.83). Two items assessing positive feelings about test results loaded together to create a third subscale (*α* = 0.73). Another two items assessing positive feelings loaded on a separate factor and were averaged to create a satisfaction with family communication and support subscale (*α* = 0.86).

Genes were categorized based on penetrance and cancer type associations. *BRCA1, BRCA2, PALB2, TP53, PTEN, CDH1*, and *STK11* were categorized as high‐risk breast cancer genes because breast cancer risks are high enough that guidelines recommend the option of a bilateral mastectomy. *ATM* and *CHEK2* were categorized as moderate‐risk breast cancer genes because they warrant breast MRIs. Genes predominantly associated with ovarian or other cancer risks (e.g., *RAD51C*, *RAD51D*, *BRIP1*, *BARD1*) and Lynch syndrome–associated genes were categorized as “other actionable hereditary cancer genes.”

Participants were categorized into “accurate recall” or “inaccurate recall” groups by comparing their self‐reported genetic test result from their baseline survey to the confirmed GPV on their test report. Those categorized into “accurate recall” correctly reported both the gene(s) and type of result(s) (i.e., positive or VUS) as outlined on the test report. Participants with a confirmed GPV in more than one gene were coded as “accurate recall” only if they indicated both the gene and result type for all GPVs associated with medical management guidelines; otherwise, they were coded as “inaccurate recall”.

### 2.3 Statistical Methods

Bivariate analyses compared those with accurate versus inaccurate recall through independent samples *t*‐tests for continuous variables and chi‐square tests for categorical variables. Demographic, clinical, and psychosocial variables with a *p*‐value below 0.1 were included in backward likelihood ratio logistic regression modeling to determine which were associated with inaccurate recall when adjusting for other variables. SPSS version 29 was used for data analysis and an *α* ≤ 0.05 determined statistical significance in the final regression model. Frequencies were calculated to further characterize the “inaccurate recall” group by calculating the percentage who failed to recall being tested and the percentages who failed to accurately recall important details of their test result.

## 3 Results

Of 807 eligible participants, 799 were recruited through ICARE, 7 through the Tennessee State Cancer Registry, and 1 through the Florida State Cancer Registry. The majority were non‐Hispanic white (91.1%), female sex at birth (93.4%), had private health insurance (81.2%), and had obtained a college degree or higher (76.6%). Per study inclusion criteria, all GPVs were actionable, with 57% of participants having a confirmed GPV in a high‐risk breast cancer gene (i.e., *BRCA1, BRCA2, PALB2, PTEN, CDH1, STK11, TP53*), 24% in a moderate‐risk breast cancer gene (i.e., *ATM, CHEK2*), and the remainder in one of many other hereditary cancer risk genes (see Table S1), Recall of GPV(s) was accurate for 716 (88.7%) participants. Demographic, clinical, and psychological variables are shown in Table 1, categorized by accurate and inaccurate recall. Bivariate analyses comparing these groups revealed statistically significant differences based on insurance, education, income, personal history of cancer, and type of cancer risk gene, as well as average age at the time of the survey, scores on the health literacy screen, hereditary cancer knowledge, cancer worry, MICRA distress, MICRA positive emotions about test results, and MICRA satisfaction with family communication and support (all *p*‐values < 0.05; see Table 1).

**TABLE 1:** Comparison of participants' demographic and psychosocial variables based on accurate recall.

	Accurate recall of GPV (*N* = 716)	Inaccurate recall of GPV (*N* = 91)	*p*
Non‐Hispanic White	657 (92%)	78 (86%)	0.057
Female sex at birth	573 (94%)	72 (89%)	0.071
Private insurance	589 (82%)	66 (73%)	0.025
Highest level of education			
No college	53 (7%)	20 (22%)	< 0.001
Some college	103 (14%)	12 (13%)	
Graduated college	294 (41%)	36 (40%)	
Professional or graduate school	263 (37%)	23 (25%)	
Prefer not to answer	3 (0.5%)	—	
Household income			
Less than $50,000	72 (10%)	22 (24%)	< 0.001
$50,000–$99,999	168 (23%)	26 (29%)	
$100,000–$149,999	179 (25%)	18 (20%)	
$150,000 and above	226 (32%)	13 (14%)	
Prefer not to answer	71 (10%)	12 (13%)	
Personal history of cancer	331 (46%)	59 (65%)	< 0.001
GPV gene categorization (all actionable)			
High‐risk breast cancer genes^a^	436 (61%)	28 (31%)	< 0.001
Moderate‐risk breast cancer genes^b^	182 (25%)	25 (19%)	
Other actionable hereditary cancer genes	98 (14%)	46 (50%)	
	**Mean (SD)**	**Mean (SD)**	
Average age at testing	46 (13)	50 (12)	0.008
Average no. of years since first tested (SD)	3.7 (3.9)	4.2 (3.6)	0.231
Health literacy (scale 1–4)	3.5 (0.6)	3.4 (0.7)	0.006
Hereditary cancer knowledge (score 0–10)	6.8 (2.0)	4.9 (2.2)	< 0.001
Cancer worry (scale 1–5)	3.1 (0.9)	2.9 (0.9)	0.031
MICRA distress (scale 0–3)^c^	0.90 (0.8)	0.65 (0.7)	0.007
MICRA uncertainty (scale 0–3)^c^	0.87 (0.8)	0.85 (0.8)	0.797
MICRA positive emotions about test result (0–3)^c^	0.58 (0.8)	0.97 (0.9)	< 0.001
MICRA satisfaction family comm & support (0–3)^c^	1.96 (1.1)	1.54 (1.1)	< 0.001

^a^ For the purpose of this analysis, those with a GPV in *BRCA1, BRCA2*, *PALB2*, *CDH1*, *PTEN*, *TP53*, and *STK11* were categorized as high‐risk breast cancer genes even if they also had a GPV in a moderate‐risk breast cancer gene or other actionable hereditary cancer gene.

^b^ Moderate‐risk breast cancer genes include *CHEK2* and *ATM*.

^c^ The 22 who did not recall being tested were not initially given the MICRA questions due to branching logic within the survey, but 10 of the 22 completed these questions after being reminded of their test result by the study team, leaving 12 missing responses for MICRA subscales.

Results of the backward logistic regression model are shown in Table 2. After adjustment for other factors, participants with a personal history of cancer had more than twice the odds of inaccurate recall compared to those without a cancer history (OR = 2.24, 95% CI 1.23–4.08). Those without a college degree also had higher odds of inaccurate recall than those with a college degree or higher (OR = 2.42, 95% CI 1.12–5.20). Greater hereditary cancer knowledge was associated with lower odds of inaccurate recall (OR = 0.67, 95% CI 0.58–0.78), as were higher distress scores (OR = 0.65, 95% CI 0.43–0.98) and greater satisfaction with family communication and support related to testing (OR = 0.62, 95% CI 0.47–0.82). In contrast, more positive emotions about the test result were associated with higher odds of inaccurate recall (OR = 1.63, 95% CI 1.17–2.26). Independent of these psychosocial and sociodemographic factors, individuals with GPVs in moderate‐risk breast cancer genes (*ATM, CHEK2*) and high‐risk breast cancer genes (*BRCA1, BRCA2, PALB2, TP53, PTEN, CDH1, STK11*) had substantially lower odds of inaccurate recall compared with participants with GPVs in other actionable hereditary cancer genes (OR = 0.18, 95% CI 0.08–0.41 and OR = 0.18, 95% CI 0.09–0.34, respectively). The model had acceptable fit based on the Hosmer–Lemeshow test, demonstrated a specificity of 98.4% and a sensitivity of 23.5%, and accounted for a moderate portion of the variability in inaccurate recall (Nagelkerke *R* ^2^ = 0.335).

**TABLE 2:** Multivariable logistic regression results of factors associated with inaccurate recall of pathogenic or likely pathogenic variant test results (*N* = 711).

Predictor	*B*	SE	OR	95% CI	*p*
Personal history of cancer (yes vs. none)	0.805	0.307	2.24	1.23–4.08	0.009
No college degree vs. college graduate	0.882	0.391	2.42	1.12–5.20	0.029
Hereditary cancer knowledge (per 1‐point increase)	−0.396	0.074	0.67	0.58–0.78	< 0.001
MICRA distress (per 1‐point increase)	−0.434	0.210	0.65	0.43–0.98	0.039
MICRA family communication/support (per 1‐point increase)	−0.476	0.140	0.62	0.47–0.82	< 0.001
MICRA positive emotions (per 1‐point increase)	0.486	0.167	1.63	1.17–2.26	0.004
Moderate‐risk breast cancer gene (*ATM, CHEK2* vs. other non‐breast cancer genes)	−1.706	0.409	0.18	0.08–0.41	< 0.001
High‐risk breast cancer gene (*BRCA1/2, PALB2*, etc. vs. other non‐breast cancer genes)	−1.729	0.328	0.18	0.09–0.34	< 0.001
Constant	1.540	0.590	4.67	—	0.009

*Note:* Model fit: Cox & Snell *R* ^2^ = 0.157; Nagelkerke *R* ^2^ = 0.335; Hosmer–Lemeshow: *χ* ^2^(8) = 3.59, *p* = 0.892; Sensitivity = 23.5%; Specificity = 98.4%.

We identified several different ways in which participants inaccurately recalled their results as shown in Table 3. Notably, 22 of the 91 (24%) who inaccurately recalled failed to remember being tested at all, whereas the remainder failed to recall various details about their test results that could impact their CRM.

**TABLE 3:** Breakdown of the 91 respondents who inaccurately recalled their germline pathogenic variant (GPV).

Inaccurate recall category	Respondents who inaccurately recalled their GPV, *N* (%)
Did not recall being tested	22 (24%)
Recalled having a GPV but did not know the gene	22 (24%)
Recalled having a GPV in the wrong gene	18 (18%)
Recalled having a VUS when they had a GPV	9 (10%)
Recalled having a hereditary cancer condition, but not which gene had the GPV (i.e., Lynch syndrome or *BRCA*)	9 (10%)
Recalled being tested but did not remember their result (i.e., failed to remember both the gene and type of result)	8 (9%)
Recalled testing negative when they had a GPV	2 (2%)
Recalled having a second GPV when the second variant was a VUS	1 (1%)

## 4 Discussion

To our knowledge, this is the largest published study to evaluate genetic test result recall among individuals with confirmed GPVs in at least one of 21 clinically actionable inherited cancer genes, each of which has clinical guidelines that would alter CRM recommendations (albeit in different ways depending on each specific gene). Factors associated with greater odds of inaccurate recall included a personal history of cancer, having a GPV in a non‐breast cancer gene (compared to those with a GPV in a high‐ or moderate‐risk breast cancer gene), lower educational attainment, more positive emotional reactions and lower levels of distress related to the test result, lower hereditary cancer knowledge, and lower satisfaction with family communication and support related to the result.

The rate of inaccurate recall of test results in our study is lower than that reported by a smaller study of 36 cancer patients, of whom 31% did not recall having germline genetic testing; and even among those who did recall testing, 44% did not remember the gene or type of result [8]. Consistent with our findings, Wing et al. [8] reported that greater hereditary cancer knowledge was associated with more accurate recall. Outside the hereditary cancer context, *APOE* genotype recall is positively associated with higher education [16], which is consistent with our findings.

Among patients newly diagnosed with cancer, high anxiety has been shown to be significantly associated with low information recall [6]. It is therefore possible that anxiety associated with a cancer diagnosis explains our findings of lower recall among those with a personal history of cancer. Alternatively, germline test results may be more easily overlooked or forgotten in the context of a cancer diagnosis due to the vast amount of information provided throughout cancer diagnosis and treatment.

Our findings that positive emotional reactions and lower distress related to test results were associated with inaccurate recall of those results are consistent with findings from a study of patients at high risk for melanoma where positive emotions or a lack of perceived threat were negatively associated with the ability to recall information [17]. Furthermore, evidence suggests that memory is enhanced more for negative experiences than positive ones [18] and distress may make the information more salient and memorable.

To our knowledge, an association between inaccurate recall and lower satisfaction with family communication and support has not been previously reported. Although this relationship highlights a potential role for family dynamics in memory reinforcement and cascade testing efforts, more work is needed to better understand the implications this finding may have.

Notably, recall was substantially better among carriers of high‐ or moderate‐risk breast cancer genes compared with those carrying GPVs in other actionable hereditary cancer genes. To our knowledge, no prior studies have directly compared recall of genetic test results across specific hereditary cancer genes. It is possible that greater public familiarity or clinical emphasis surrounding hereditary breast cancer genes may contribute to the higher recall observed in our sample.

Our study has several strengths, including being among the largest studies to report on recall of genetic test results among individuals, all of whom have a confirmed, clinically actionable GPV in a variety of genes, inclusive of both males and females as well as those with and without various types of cancer. Additionally, we were able to evaluate psychosocial correlates of inaccurate recall that had not been well characterized previously. Finally, we provided a detailed typology of recall errors and identified unique psychosocial predictors.

Despite these strengths, there remain some limitations, including the high socioeconomic status and education level of our participants, which limits the generalizability of our findings. Furthermore, given that inaccurate recall was more common among individuals without a college degree, our findings likely underestimate the true prevalence of inaccurate recall in the broader population. Additionally, while we identified several important factors associated with recall accuracy, the relatively low event rate of inaccurate recall limits the predictive power of our model. Moreover, the cross‐sectional design of the study precludes causal inference. Finally, we could not assess for differences in recall based on family history, as family history data were incomplete.

In addition to study limitations, it is important to note how various types of recall errors have different implications for clinical management. Serious consequences may arise for individuals who do not recall having genetic testing, those who recall a negative result when the test was positive, or those who recall a VUS rather than a positive result, as these scenarios may prevent them from receiving proper high‐risk cancer management and therefore may contribute to a higher incidence of cancer or cancer being diagnosed at a later stage. However, other forms of misremembering could also lead to harm. For example, participants who believed they had a GPV when they did not could undergo unnecessary screening or risk‐reducing surgeries. Similarly, recalling the wrong gene could result in inappropriate management if documented inaccurately. In contrast, recalling having testing but not the specific gene may be less harmful, as long as the provider and/or patient seek out a copy of their test result and associated CRM guidelines. Finally, recalling the hereditary cancer syndrome, but not the specific gene, may have less severe consequences. Nevertheless, differences in cancer risks and CRM guidelines among Lynch syndrome and *BRCA* genes mean that even partial recall can still contribute to suboptimal care unless results are readily available and verified.

Ultimately, our findings have several clinical implications, as many of the factors associated with accurate recall that we identified could be targeted through interventions. For example, patients who seem emotionally reassured or express positive feelings about their test result may not necessarily understand the full implications and may be less likely to remember their results accurately. Moreover, strategies such as teach‐back methods, the use of open‐ended questions, limiting information overload, and providing personalized information sheets or copies of test results might represent strategies to reinforce understanding and memory retention [19, 20, 21]. Finally, for some patients, a single disclosure of test results may be insufficient for long‐term retention. Because memory degrades over time and emotional framing affects recall, reinforcement strategies such as follow‐up visits, written summaries in plain language, digital reminders, periodic re‐disclosure tools, and easy access information through patient portals are strategies that could be considered. Our findings also highlight how important it is that providers obtain copies of patients' germline test results before making CRM decisions to ensure risk appropriate care.

Additional studies are needed to explore scalable interventions aimed at improving recall, particularly among populations at higher risk of inaccurate recall and inclusive of more diverse populations to understand broader patterns in genetic test result recall. Furthermore, it remains important to investigate whether inaccurate recall is associated with failure to follow recommended CRM strategies or engagement in inappropriate interventions.

In conclusion, we have identified several predictors of inaccurate recall among a population of patients with GPVs in inherited cancer susceptibility genes in whom ongoing CRM is warranted. Importantly, even in our highly educated sample, over 11% failed to accurately recall their result, underscoring that recall challenges are not confined to low‐literacy populations. Consequently, our findings highlight the importance of identifying predictors of inaccurate recall given the implications for CRM. These findings highlight the need for longitudinal, patient‐centered communication strategies to reinforce critical genetic information over time and the importance of obtaining copies of germline genetic test reports to verify accuracy.

## Author Contributions

**Deborah L. Cragun:** conceptualization, funding acquisition, investigation, methodology, formal analysis, supervision, writing – original draft. **Brittany F. Sears:** conceptualization, formal analysis, investigation, methodology, writing – original draft. **Karishma Prakash Bharwani:** investigation, methodology, writing – review and editing. **Anne E. Weidner:** data curation, investigation, methodology, project administration, writing – review and editing. **Jason W. Beckstead:** formal analysis, writing – review and editing. **Tuya Pal:** conceptualization, funding acquisition, investigation, methodology, supervision, writing – review and editing.

## Ethics Statement

This study received approval from Vanderbilt (IRB #202215), the Tennessee Department of Health IRB (IRB# 2021‐0281), and the Florida Department of Health IRB (IRB# 2023‐022‐VBU).

## Conflicts of Interest

B.F.S. is currently a full‐time, salaried employee of Ambry Genetics, but most of the work on this manuscript occurred before she began working with Ambry.

## Supporting information


**Table S1:** cam471689‐sup‐0001‐SupplementaryTable1.docx.

## Data Availability

The data that support the findings of this study are not publicly available due to their highly identifiable nature and the sensitive health information they contain. Access to the data may be granted upon completion of all required data use agreements and institutional approvals upon reasonable request. Interested researchers should contact the corresponding author for further information.
