# Supplementary material for: Factors Associated With Inaccurate Recall of Inherited Cancer Genetic Test Results Among Individuals With Germline Pathogenic Variants
Source: Cancer Med. 2026 Mar 7;15(3):e71689. doi: 10.1002/cam4.71689 (PMC12967459; doi:10.1002/cam4.71689)
Supplement: Supplementary file 1 — Table S1: cam471689‐sup‐0001‐SupplementaryTable1.docx. [file CAM4-15-e71689-s001.docx]

**Supplemental Table 1.** Recall of inherited cancer genetic test results by gene

| Gene | GPV All participants N=817 | % of all participants | GPV Accurate recall n=716 | GPV Inaccurate recall  n=91 |
| --- | --- | --- | --- | --- |
| *APC* | 7 | 0.9% | 4 (0.6%) | 3 (3.3%) |
| *ATM* | 95 | 11.6% | 88 (12.3%) | 7 (7.7%) |
| *BARD1* | 12 | 1.5% | 9 (1.3%) | 3 (3.3%) |
| *BRCA1* | 104 | 12.7% | 102 (14.4%) | 2 (2.2%) |
| *BRCA2* | 167 | 20.4% | 155 (21.6%) | 12 (13.2%) |
| *BRIP1* | 4 | 0.5% | 2 (0.3%) | 2 (2.2%) |
| *CDH1* | 4 | 0.5% | 4 (0.6%) | 0 |
| *CDKN2A* | 9 | 1.1% | 8 (1.1%) | 1 (1.1%) |
| *CHEK2* | 112 | 13.7% | 101 (14.1%) | 11 (12.1%) |
| *EPCAM* | 1 | 0.1% | 1 (0.1%) | 0 |
| *MLH1* | 15 | 1.8% | 9 (1.3%) | 6 (6.6%) |
| *MSH2* | 21 | 2.6% | 15 (2.1%) | 6 (6.6%) |
| *MSH6* | 38 | 4.7% | 25 (3.5%) | 13 (14%) |
| *MUTYH* (biallelic) | 3 | 0.4% | 2 (0.3%) | 1 (1.1%) |
| *PALB2* | 173 | 21.2% | 162 (22.6%) | 11 (12.1%) |
| *PMS2* | 34 | 4.2% | 26 (3.6%) | 8 (8.8%) |
| *PTEN* | 3 | 0.4% | 2 (0.3%) | 1 (1.1%) |
| *RAD51C* | 5 | 0.6% | 4 (0.6%) | 1 (1.1%) |
| *RAD51D* | 3 | 0.4% | 2 (0.3%) | 1 (1.1%) |
| *STK11* | 5 | 0.6% | 4 (0.6%) | 1 (1.1%) |
| *TP53* | 11 | 1.3% | 10 (1.4%) | 1 (1.1%) |

Note: Among the 807 participants, 23 had a GPV in 2 genes, 780 in one gene, and 4 who reported a GPV had a VUS upon test report verification. Several had GPVs in a high-risk and moderate risk breast cancer genes.
